# Hospital and Institutional News

**Published:** 1920-07-03

**Authors:** 


### HOSPITAL AND INSTITUTIONAL NEWS.

### HOSPITAL SUNDAY.

Another Hospital Sunday has come and it is the hope of many that the wide publicity given to the needs of our hospitals during the last few weeks had an influence in unloosening the purse-strings of those who attended places of worship last Sunday. But it was not only church-goers and chapel-goers who were asked to give, for a street campaign, organised by the British Red Cross Society, was undertaken. Nearly 1,000 men and women, boys and girls, gave their services as collectors, and it is rumoured that their efforts were well repaid by success. The pressing need of the hospitals was brought home to the London public at every turn. Omnibus and tramway stopping-places were favourite places for the amateur beggars, and in the parks the Sunday crowds liberally responded to the appeal. In some districts carnivals and demonstrations were held, and house-to-house collections went over ground neglected by the remainder of the campaign.

### AT ST. PAUL'S AND THE ABBEY.

The Lord Mayor and the Sheriffs, as is customary, attended in state the forenoon service at Westminster Abbey, and in the afternoon they, together with members of the Common Council and a number of judges of the High Court, visited St. Paul's Cathedral for early evensong. At the Cathedral the civic dignitaries and judges were met at the west door by the Dean and Chapter, who preceded them to the choir. At St. Paul's the sermon was preached by Canon Alexander, who said that the hospitals, like our cathedrals, were living on the past, without any clear or definite policy. He dreaded even the temporary abandonment of the voluntary system on which we prided ourselves, but he did not doubt that the private wealth of the country would be sufficient to meet the crisis if men's hearts were rightly touched. Among the large contributions received by the Lord Mayor this year for the fund are the following:—Lady Strathcona, £4,000; Lloyd's Bank, £1,000; Forestal Land, Timber, and Railways Company, Limited, £525; London County Westminster and Parr's Bank, £500; Watts, Watts, and Co., Ltd., £315; Sir A. Hargreaves Brown, £300; Sir John Latta, £262 10s.; Blyth, Greene, Jourdain, and Co., Ltd., £210; and Thomas Stephens and Sons, Ltd., Bank of Australasia, Ltd., and Lieutenant-Colonel More-Nisbett, £200 each.

### THE GENERAL PRACTITIONER'S HANDICAP.

With reference to our correspondent's personal plea on p. 356, it is interesting to note the views expressed by Sir Clifford Allbutt in his recent presidential address to the B.M.A. at Cambridge. Panel practice, he remarked, for some time discredited by its hereditary taint of the club of infamous memory, and by the continual dearth of the means of its development, should, if duly provided, expand into a large, honourable, and even universal department of medical work; the terms of engagement were to be more liberal; the clockwork was complete. But how was the practitioner to rise to higher standards of modern science and skill when no means of investigating disease, or of making an intimate diagnosis, were provided for him? He was well aware that the modern methods of medicine depended on such means as *x*-rays, a battery of stains, and other cultural and bio-chemical apparatus; and not only an education in the use of these, but also the time to give to them. What should we think of a regiment of recruits called out to fight the enemy but unprovided with weapons or munitions? If the doctor were near a university centre he could get blood tested for morphological elements or for sugar or nitrogen content, excretions analysed, bacterial examinations and vaccines made, and so forth; but it was imperative that these opportunities, now confined to a few, should be universal. Moreover, if they were to be fruitful, the practitioner must consult personally with the scientist. These plants, then, and their staff must be established in all districts, together with a much larger provision of cottage hospitals. Cambridge University and its sisters were sending out every year bands of young graduates quite competent to deal with disease in all its forms, and to use refined means of diagnosis, if those means were supplied. If not, how could they be blamed if they fell into some stagnant routine? In district laboratories panel work would be paid by the Ministry of Health; for private practice a tariff of fees would be agreed upon. A considerable sum for the commencement of these local centres was contained in the still-born Budget of 1914.

### SHOULD THE DOCTOR TELL?

It is not very often that the debates at the representative meeting of the British Medical Association arouse any general public interest. But this year one of the topics under discussion touches very nearly the lives and happiness of so many people that the ventilation of it has been followed with unusual closeness by both press and public: the debate, that is, upon the obligation of professional secrecy. After conflicting views had been put forward, sometimes with vehemence, the Association's solicitor drafted a resolution which satisfied all but two of the delegates: naturally enough, a resolution which could achieve this result in connection with so controversial a question was a trifle colourless and non-committal. It was worded thus: "That having further considered the question of professional secrecy from the standpoint of the medical profession, and with special regard to venereal diseases, the representative body reiterates the opinion that the medical practitioner should not without his patient's consent voluntarily disclose information which he has obtained from such patient in the exercise of his professional duties." A further resolution to the effect that the medical profession ought to be put on the same footing as to professional secrecy with clergy, barristers, and solicitors was lost. To the best of our belief there is no statutory recognition of any such privilege in regard to the clergy: the position is that they regard themselves as bound to secrecy and will go to prison for contempt of court rather than forgo their claim. (See p. 359.)

### THE PRINCIPLE AT ISSUE

The position of the medical profession in this matter is one of great difficulty in many cases. Admittedly, if public confidence in the inviolability of consulting-room secrets is shaken, many men and women will avoid the doctor just when it is most important in their own interests and in those of others that they should have prompt and efficient treatment. The State, the medical profession, and (more reluctantly, perhaps) the public have accepted disclosure in regard to smallpox, diphtheria, and a dozen other notifiable diseases; and have even decided that in many such cases the liberty of the individual to infect others with his disease must be forcibly restricted by compulsory isolation. Having swallowed the camel, it is argued, why strain at the gnat of venereal disease? The other argument against the resolution adopted is that self-imposed silence may mean misery or death to innocent persons, and that the doctor has in such circumstances no moral right to hold his tongue. While this is true enough, the question at issue is not primarily a moral, but a practical one. The greatest good of the greatest number must be the first consideration. The number of cases in which moral suasion fails to prevent the exposure of the innocent to infection must be very small indeed. The harm done by concealment of disease if the profession gave up its obligation of secrecy would probably be many times greater. The one outstanding moral to be drawn from the discussion is that every doctor practising his profession ought to join the Medical Defence Union or one of the similar associations.

### GRADED MEDICAL SERVICE.

WITH the idea of providing first-class medical attention for those employees of limited means on the staff of Messrs. Thomas De La Rue and Company, and its associate companies, Mr. Stuart De La Rue has arranged an extension of the medical service provided by his company so as to include an organisation of eminent consulting physicians and surgeons who would agree to treat privately patients sent by the company at fees reduced in such a way that individuals should pay only according to their ability to pay. We have received full particulars of this scheme which is now being brought into operation and hope to deal with it more fully in a subsequent issue.

### DR. MARIE STOPES AND BIRTH CONTROL.

WE have received a letter from Dr. Marie Stopes, who is a member of the Birth-Rate Commission, on the subject of the problem of so-called birth control. She points out that eleven members of the Commission signed Reservation III of the Report stating that medical evidence had been given condemning entirely all methods of control of conception. Hence she sent a letter to each signatory of the reservation, in which she said she considered the question of the right use of sound hygienic methods of birth control one of the very greatest importance to the human race, and challenged them to indicate any medical facts in the evidence upon which they based their sweeping statements. Dr. Stopes, in reply, says that not one of the recipients quoted a page or paragraph containing the medical evidence which they postulate, but several did reply that their objection was a moral one. As the reservation explicitly stated that its basis was a medical one, the confusion of thought of the signatories was evident. The counter-reservation (Reservation IV), however, is true—namely, that much evidence was given to show that the use of the best contraceptives was much *less* harmful than any other course open to large numbers of people. In conclusion, Dr. Stopes declares there is urgent need that on a subject of such immense racial and individual concern, a Royal Commission, which shall hear full and competent evidence and much evidence from poor *women*, should be set up.

### THE NEW PRESIDENT OF THE BRITISH HOSPITALS ASSOCIATION.

SIR ARTHUR STANLEY'S well-known smile is a great asset in these trying times, and it was greatly in evidence when, amidst general enthusiasm and plain indication of popularity, he took the chair as the new President of the British Hospitals Association at the annual meeting at St. Thomas's Hospital on June 25, in succession to Viscount Sandhurst, whose resignation was regretfully announced and received. The meeting was poorly attended, probably because of the formal and unexciting nature of the published agenda, but the proceedings were very interesting, and it is evident that the British Hospitals Association is going ahead with great strides. The idea of areas or regions is developing rapidly, and, geographically, is following the model of the Ministry of Pensions and other public bodies. There are to be twelve regions—Scotland, Northern Counties, North-Western (including Lancashire), Yorkshire, Wales, West Midlands, East Midlands, Eastern, South-Western, London (with several adjacent counties), and two regions in Ireland, the latter provision having, we believe, no political significance. The idea is that some prominent hospital man in each region will be asked to get together a meeting of hospital representatives, who will send a chosen member to the General Council of the British Hospitals Association. Lord Winterton pointed out that Parliament was always impressed by the representative character of an association, and if the British Hospitals Association plays the part expected in the future in respect of State aid or State control and other matters, the widening of its electoral basis is very much in the right direction.

### THE COLLEGE AND THE ASSOCIATION.

SIR ARTHUR STANLEY announced that, in reference to proposed legislation as to hours of employment, the Ministry of Labour desired to obtain the opinion of some body representing hospitals as a whole, and that it would probably seek the views of the Association. It was then decided that a conference shall be arranged between the College of Nursing and delegates of the Association, and Mr. C. E. Leonard Lyle, M.P., the Rev. G. B. Cronshaw, Mr. G. Q. Roberts, and Mr. Frank Hazell, gentlemen whose choice in this respect requires no explanation, were appointed to meet the College authorities and to decide upon the attitude suggested to hospitals as to hours of duty of nurses. No directions were given to the delegates, but from the general trend of the debate it was gathered that a fifty-six-hour week was most in favour. In any case, the plan decided upon will be circulated amongst members of the Association.

### PATIENT'S FEES AT THE LONDON HOSPITAL.

As was generally predicted at the time, the 10s. per week voluntary payment by patients proposed by the London Hospital some months ago is now found to be not worth the trouble of collection, and it has now been decided that, with the exception of the very poor and of young children, the charge shall be 21s. a week in all cases, exemption only to be granted on the production of satisfactory and written evidence from some responsible person of inability to pay. The worst of the flat rate principle, when applied in a rigid manner, is that many who could pay less escape altogether and many who could pay more make a saving; in respect of the latter class it is proposed at the London that a voluntary additional contribution should be suggested on discharge. Naturally, accident and emergency cases will be admitted immediately and arrangements made as to payment at the earliest possible moment, but in respect of other patients two weeks' payment in advance will be required. Mr. Morris is reported to have said that "It is a pretty awful thing for us to have to do this, and everybody in the hospital hates the thought of it." We do not quite understand such extreme repugnance, for in each case the family budget is being saved a considerable sum, which means that the hospital is making a gift of money to the patient or the patient's relatives. Medical attendance, the use of valuable apparatus, nursing, and a host of other things are still given without payment. In any case, the 21s. asked by the London is only about a quarter of the average weekly cost of the bed.

### THE END OF THE "NATIONAL FOOD JOURNAL."

The final number of the *National Food Journal* appeared on June 9, having since its appearance on September 12, 1917, achieved its third volume and fifty-third number. With the cessation and simplification of food control the usefulness of the journal has become rather small, and in the interests of economy we approve of its withdrawal. But in the early days of control there is no doubt that it was a most useful organ, containing, as it did, summaries of the Food Controller's Orders in handy and popular form. In spite of an extensive free distribution, latterly reaching 40,000 copies of each issue, the cost of the journal has been less than was expected. The total net cost of the fifty-three numbers has been approximately £4,700, or £89 per issue. The average proceeds from sales have been about £200 per issue.

### GOOD LUCK FOR BIRMINGHAM.

A feature of the will of Mr. William Barwick Gregoe-Colmore, of Cadogan Place, S.W., who has left the seventh millionaire estate of the current year, is that his trustees are requested to continue his subscriptions to the institutions which benefit under the will and to any others to which he was an annual subscriber, such contributions to be paid during the lives of his sisters, and of his niece, or for two years, whichever be the longer, but they may refuse to do so in their discretion. Mr. Gregoe-Colmore was a large landowner in Birmingham, and, with the exception of £500 to the Cheltenham Hospital, it is only Birmingham charities which are mentioned in the will. £500 is bequeathed to the General Hospital, £200 each to the Queen's, the General Dispensary, the Blue Coat School, the Edgbaston Blind Institution, the Deaf and Dumb Institution, the Children's Hospital, the Eye Hospital, the Orthopædic and Spinal Hospital, and the Birmingham and Midland Counties Sanatorium. £100 each is bequeathed to the Dental Hospital, the Lying-in Charity, the Homœopathic Hospital, the Ear and Throat Hospital, the Skin and Lock Hospital, and the Women's Hospital. In addition to the above comprehensive list of charities, Mr. Gregoe-Colmore left handsome legacies to his servants. The estate and legacy duties payable on the property will amount to about £362,800.

### ROBBING THE HOSPITALS.

A man, described as an engineer, was remanded at the Guildhall last week on a charge of stealing a collection-box belonging to St. Bartholomew's Hospital from a shop in New Oxford Street. The detective who arrested the defendant said that he admitted the offence and confessed that he had broken up the box and thrown it away. He further admitted that he had obtained the headings of hospital collecting forms, stuck them on sheets of foolscap, and collected considerable sums which he spent on himself. In an attaché case belonging to the defendant were found six lists showing that he had received some £50, and amongst the contents was another hospital collecting-box. The man stated that he was wanted for offences in Birmingham, Norwich, and Brighton. No carelessness on the part of hospitals was disclosed, but it goes without saying that collection-boxes and lists should be entrusted only to persons who are known to be trustworthy.

### THE AMERICAN HOSPITAL ASSOCIATION.

A permanent home for the American Hospital Association was opened last month at 22 East Ontario Street, Chicago. The town was chosen because many other American hospital activities are centred there, and the office of the *Modern Hospital* newspaper is in the same building, as are the headquarters of the Catholic Hospital Association, while the American Medical Association and the American College of Surgeons are housed near by. A library and service bureau for hospitals is to be established to fill and distribute information. The Association is conducting a national survey of Hospital Social Service departments through a special committee in association with the bureau on dispensaries and community relations. Dr. A. R. Warner is the executive secretary.

### AN EXAMPLE FROM SOUTHPORT.

The Southport Trades Council has received a deputation from the Southport Infirmary to gain increased support for the institution and to offer representation on the Board. The matter has been passed on to a committee of the whole Councils. Meantime the Vulcan Works has, by an organised collection, increased its subscription from £40 to £400, and the Chamber of Trade has decided to send a representative to help the infirmary. Mr. J. H. Shaw, the Secretary, has explained the position; and at the recent deputation Mr. H. Brooke, J.P., remarked that since the National Insurance Act became law the interest of the working men has declined. There are signs, however, that their interest can be revived, and the example of the Vulcan Works shows what can be done by a little good-will and organisation.

### A WELL-KEPT SECRET.

Southwark guardians were somewhat astonished when they learnt that an aged woman who had been in their institution for over twelve months was found with a piece of waist flannel in which were a deposit receipt for £320 on the London Bank of Australia and £5 13s. 2½d. in cash. She had previously been in Lambeth Infirmary, and the lady's secret had not been discovered by the many officers who, from time to time, had examined her clothing and superintended her bathing. At length one lynx-eyed nurse at Newington Institution discovered her secret when she was having a bath. The holder of the wealth was formerly a professional beggar living in Mint Street in the Borough. The money was left to her by a brother. Having found the cash and the receipt, the guardians are anxious to take from it the cost of her maintenance in the institution, but, as the old lady is obstinate and refuses to sign any documents, it will be a difficult task.

### THE HOSPITAL SITUATION IN ABERDEEN.

Little progress can be reported in the scheme for the erection of a central general hospital for Aberdeen on one site near but not inside the city. We have reported the arguments in favour more than once before now, but in Aberdeen, as in Bristol, the closer the project of amalgamation is approached, the more difficulties and hesitations appear. Money perhaps is the chief lack in Aberdeen at present, and the ambitious plan for the co-ordination of the Royal Infirmary and Royal Hospital for Sick Children has received only £23,000 odd so far. Meantime, the waiting-list and the number of beds at the Royal Infirmary are about equal, and Sir Thomas Burnett, Bart., of Crathes Castle, one of the directors of the children's hospital, has declared it to have "the worst accommodation of any hospital in Aberdeen," a verdict which he said was proved to be just because the narrow passages and steep stairs nearly converted a recent fire into a grave catastrophe. A scheme for raising money has been proposed by Sir James M'Taggart, but as its details were not disclosed at the time when Sir Thomas lamented the shortage of money, we cannot unfortunately report the one constructive proposal which has been made.

### A HOUSE CENSUS IN BERMONDSEY.

Bermondsey Borough Council is examining all the house property in the borough, with a view to compelling the owners to put it in a reasonable state of repair. The staff was insufficient to carry out such a gigantic task and do the ordinary work of the borough at the same time. To overcome this difficulty the Council decided to appoint two assistant medical officers and four temporary sanitary inspectors to assist Dr. King Brown, the medical officer. Already 162 houses have been scheduled for repairs, and, as only the owners of about twenty have decided to do the necessary work, the Council have instructed their borough surveyor to do the requisite repairs. The cost will be recovered from the owners, who, if they decline to pay, can, under the Housing and Town Planning Act, be summoned for the amount due in a police court. The task is a great one, both for the medical and sanitary staff and for the borough surveyor's department. There is a tremendous lot of dilapidated property in the borough, and as the officers continue their survey the number of houses placed on the list for repairs will gradually mount.

### INFIRMARIES AND MEDICAL STUDENTS.

St. Pancras Guardians have decided to adopt a proposal of their Infirmary Committee whereby the material at their infirmary will be available for the students at the London School of Medicine for Women. As previously noted, Paddington Infirmary is to be used by the students of St. Mary's Hospital, and, although details of the full arrangements made at St. Pancras are lacking at the moment, no doubt this will include visits of consultants and should tend to develop the work which Poor-Law Infirmaries have managed to carry out in the past with small medical and nursing staffs.

### THIS WEEK'S DRUG MARKET.

Business is worse rather than better. Generally speaking prices continue to sag and very little buying is going on. Japanese camphor has had another substantial fall in price and can now be bought at less than one-third the figure quoted six months ago. Menthol has also dropped heavily during the week. These, however, are somewhat exceptional articles, since in the majority of cases where prices have moved downwards, the movement has been slow. In the absence of large scale business, prices in many cases are merely nominal.

